# Anti-CD123 CAR-T therapy combined with autologous SCT and venetoclax maintenance in refractory BPDCN ineligible for allogeneic transplantation: a case report and review of the literature

**DOI:** 10.3389/fimmu.2026.1922400

**Published:** 2026-07-31

**Authors:** Fan Yang, Danyang Li, Yang Lei, Rui Liu, Zhonghua Fu, Lixia Ma, Biping Deng, Xiaoyan Ke, Kai Hu

**Affiliations:** 1 Department of Lymphoma and Myeloma Research Center, Beijing GoBroad Hospital, Beijing, China; 2 Cytology Laboratory, Beijing Gobroad Boren Hospital, Beijing, China

**Keywords:** ASCT, blastic plasmacytoid dendritic cell neoplasm, CD123 CAR-T, IEC-HS, venetoclax

## Abstract

Blastic plasmacytoid dendritic cell neoplasm (BPDCN) is an aggressive hematologic malignancy with limited therapeutic options for patients ineligible for allogeneic hematopoietic stem cell transplantation (allo-HSCT). While CD123-targeted therapies and CAR T-cell infusion have shown promise, achieving durable remission without consolidative transplantation remains challenging. We report a pioneering “triple-integrated” consolidation strategy in a 55-year-old male with relapsed/refractory BPDCN and central nervous system involvement who lacked a suitable HLA-matched donor. After achieving a complete metabolic response with persistent bone marrow minimal residual disease (MRD) following Hyper-CVAD chemotherapy, the patient underwent high-dose conditioning and autologous stem cell transplantation (ASCT) sequentially followed by autologous CD123 CAR T-cell infusion (1.74×10^6^/kg). The clinical course was complicated by Grade 3 cytokine release syndrome and suspected immune effector cell-associated HLH-like syndrome (IEC-HS), which were successfully managed with glucocorticoid and emapalumab. Notably, ASCT served as a “hematopoietic rescue” for CAR-T-induced prolonged cytopenia. To prevent late clonal escape, maintenance therapy with the BCL-2 inhibitor venetoclax was initiated post-transplant. The patient achieved sustained MRD-negative CR with a disease-free survival exceeding 13 months. This multimodal paradigm—combining intensive cytoreduction, targeted immunotherapy with marrow rescue, and molecular maintenance—provides a feasible and potentially curative alternative for BPDCN patients in the “no-donor” setting.

## 1 Background

Blastic plasmacytoid dendritic cell neoplasm (BPDCN) is an ultra-rare, aggressive hematologic malignancy derived from plasmacytoid dendritic cell precursors. It is characterized by a hallmark immunophenotype—including CD4, CD56, TCL1, and robust expression of CD123—and a dismal prognosis, with a median overall survival (OS) of only 8–14 months under conventional chemotherapy (1–3).

While achieving first complete remission (CR1) followed by allogeneic hematopoietic stem cell transplantation (allo-HSCT) remains the established curative standard, a significant therapeutic impasse exists for patients who are ineligible for allo-HSCT due to advanced age, comorbidities, or the lack of a suitable HLA-matched donor (4–8). Recent advances in CD123-targeted therapies, such as tagraxofusp and pivekimab sunirine (PVEK), have improved response rates; however, these agents frequently serve as a temporary “bridge” to transplantation, with limited long-term durability when used as monotherapy (9–12).

Anti-CD123 chimeric antigen receptor (CAR) T-cell therapy has emerged as a promising “living drug” capable of profound molecular debulking in BPDCN (13–17).Nevertheless, the optimal strategy for consolidating CAR-T-induced remissions in the “no-donor” setting remains undefined, particularly regarding the prevention of late clonal escape.

In this report, we describe a novel “triple-integrated” consolidation strategy for a patient with relapsed/refractory (R/R) BPDCN lacking an allogeneic donor. This multimodal approach utilized high-dose conditioning followed by autologous stem cell transplantation (ASCT) and sequential anti-CD123 CAR-T infusion, followed by maintenance therapy with the BCL-2 inhibitor venetoclax. This paradigm resulted in a durable disease-free survival (DFS) exceeding 13 months, offering a potential curative-intent alternative for transplant-ineligible BPDCN patients.

## 2 Case presentation

### 2.1 Clinical presentation and diagnosis

A 55-year-old previously healthy male presented with painless cervical and axillary lymphadenopathy associated with disseminated violaceous, non-blanching skin plaques on the neck and upper arms. Systemic symptoms (B-symptoms) were absent. Ultrasonography confirmed generalized lymphadenopathy (largest node: 3.3×1.2cm in the right inguinal region).

Biopsy of an axillary lymph node revealed a malignant hematopoietic infiltration. Immunohistochemistry (IHC) demonstrated a profile diagnostic of BPDCN: CD123+, CD4+, CD56+, CD43+, TCF4+, TCL1A+, TdT+, and CD33+, while lineage-specific markers (CD3, CD5, CD19, CD20, MPO, and CD34) were negative. The Ki-67 index was 40%–60%, and BCL-2 was strongly expressed (~100%).

### 2.2 Baseline evaluation and frontline therapy

Baseline evaluation (**Supplementary Table 1**) revealed mild pancytopenia. Bone marrow (BM) aspirate showed 26% BPDCN blasts with a normal 46,XY karyotype. Flow cytometry of the cerebrospinal fluid (CSF) indicated 10.54% CNS involvement, despite a normal cranial MRI. PET-CT demonstrated multi-focal hypermetabolic lesions involving lymph nodes (supra- and infra-diaphragmatic), liver, spleen, BM, and skin.

Frontline therapy comprised alternating Hyper-CVAD (A/B) cycles combined with intrathecal (IT) therapy. Following four weeks of weekly IT chemotherapy, cerebrospinal fluid (CSF) analysis confirmed complete clearance of initial involvement. Maintenance IT prophylaxis was continued at 1–2 doses per cycle thereafter. After 9 cycles, he achieved a complete response (CR) and CNS clearance. However, BM minimal residual disease (MRD) remained persistent (0.98%). In the absence of a suitable HLA-matched or haploidentical donor for allogeneic HSCT, we pursued a novel consolidation strategy comprising ASCT followed sequentially by autologous CD123 CAR T-cell therapy. This regimen utilized high-dose conditioning followed by autologous stem cell rescue (Day 0) and timely anti-CD123 CAR-T infusion (Day +2) to achieve deep marrow eradication.

### 2.3 Anti-CD123 CAR-T therapy combined with autologous stem cell transplantation

In December 2024, the patient was admitted to our institution for a comprehensive pre-transplant evaluation; his baseline clinical characteristics are summarized in **Supplementary Table 1**. Following mobilization with methotrexate and cytarabine (MA), peripheral blood stem cells (PBSCs) were successfully harvested, yielding 3.365×10^6^ CD34+ cells/kg.

**Figure 1:**
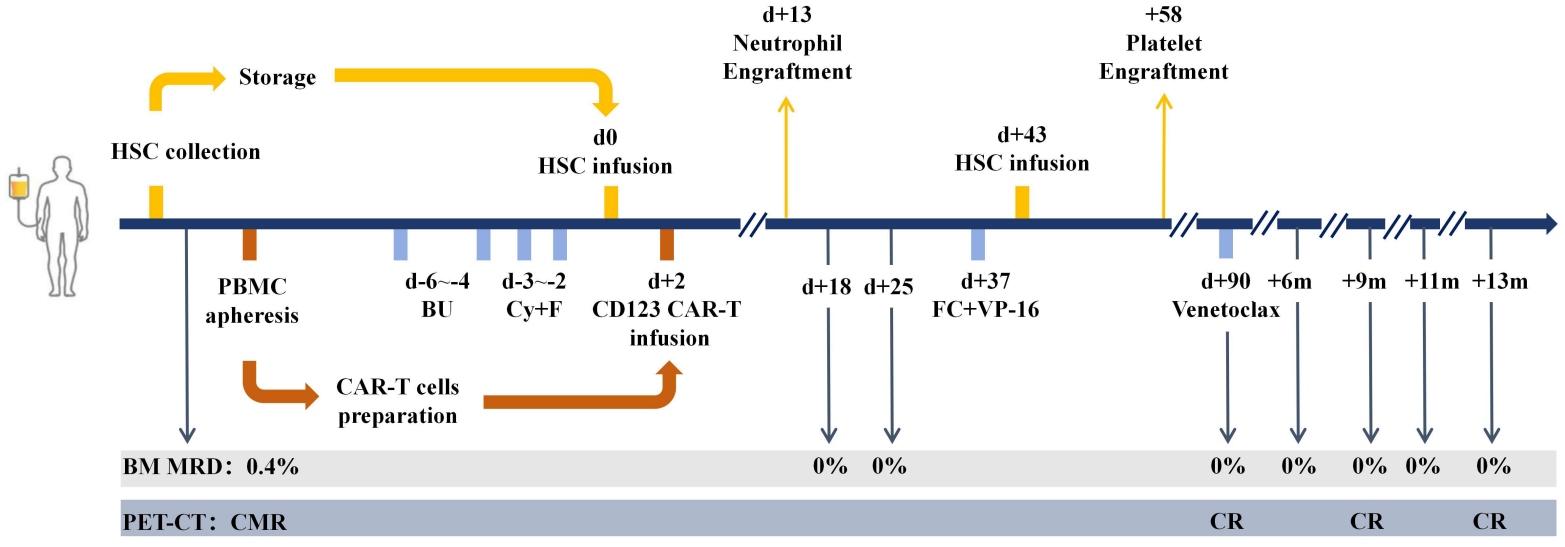
Therapeutic schema of the integrated ASCT and CD123 CAR T-cell regimen. HSC, hematopoietic stem cell; PBMC, peripheral blood mononuclear cells; BU, Busulfan; CY, Cyclophosphamide; F, fludarabin; FC+VP-16, Fludarabine, Cyclophosphamide and Etoposide; MRD, Minimal residual disease; CMR, Complete metabolic response; CR, complete remission.

The combination therapy regimen is outlined in **Figure 1**.This study was conducted as an investigator-initiated trial (IIT), with the protocol approved by the Institutional Review Board (IRB) of Beijing GoBroad Hospital (Ethics Approval No. KY2024-001-001). All procedures adhered to the ethical principles of the Declaration of Helsinki, and the patient provided written informed consent. Autologous lymphocytes were harvested via leukapheresis for CAR T-cell manufacturing. The anti-CD123 CAR construct incorporated a humanized scFv targeting CD123, a 4-1BB costimulatory domain, and a CD3ζsignaling domain, delivered via a lentiviral vector. CAR T cells were manufactured in the GMP-compliant facility of Beijing GoBroad Boren Hospital under standardized operating procedures (SOPs). Following a 7-day expansion period, the final product demonstrated a transduction efficiency of 42.5% (assessed by flow cytometry) and a cell viability of 84%.

Subsequently, the patient received a conditioning regimen consisting of busulfan (3.2 mg/kg/d, days -6 to -4), cyclophosphamide (50 mg/kg/d, days -3 to -2), and fludarabine (30 mg/m²/d, days -3 to -2). Autologous hematopoietic stem cells (2.04×10^6^ CD34+ cells/kg) were reinfused on Day 0, followed by the infusion of 1.74×10^6^/kg CD123 CAR T-cells on Day +2.

### 2.4 Toxicities and management

**Figure 2:**
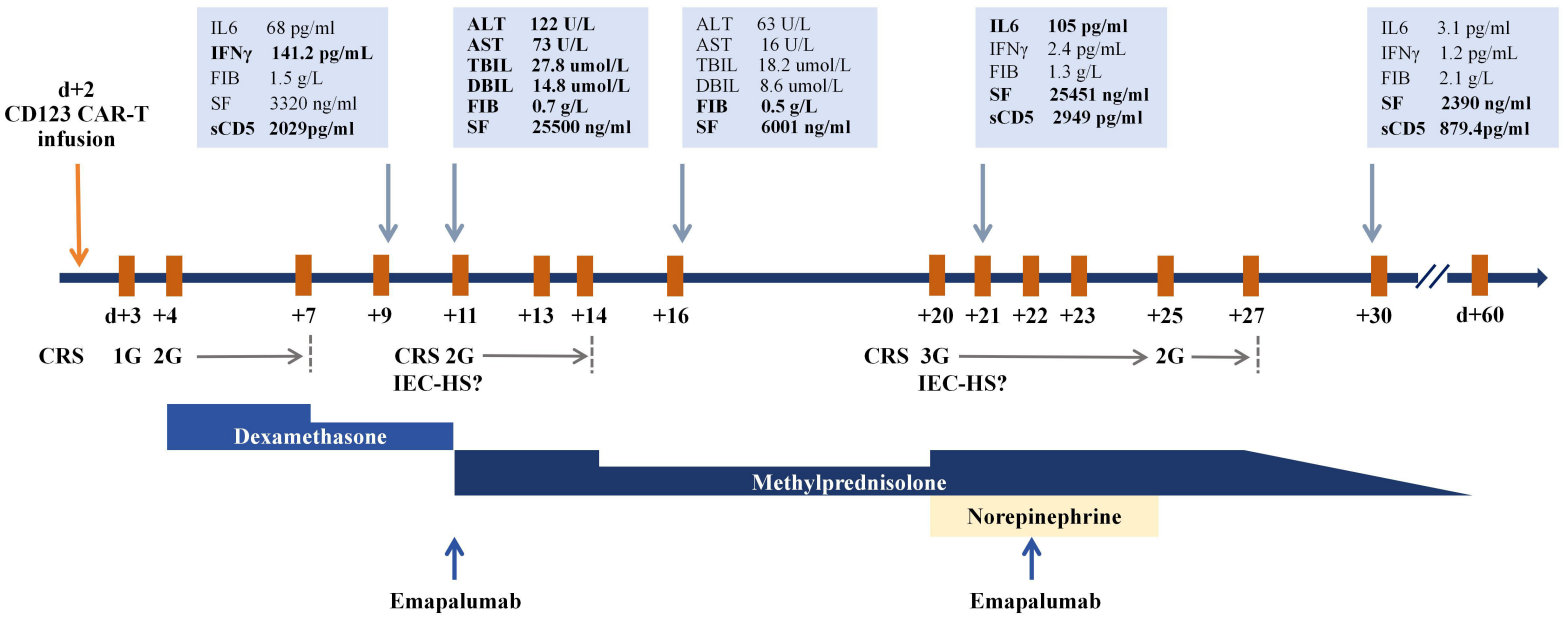
Timeline of adverse events and clinical management during the first 30 days post-transplant. CRS, cytokine release syndrome; IEC-HS, immune effector cell-associated HLH-like; IL6, interleukin-6; IFNγ, interferon; SF, serum ferritin; FIB, fibrinogen; sCD25, soluble CD25; ALT, alanine aminotransferase; AST, aspartate aminotransferase; TBIL, total bilirubin; DBIL, direct bilirubin.

On Day +3, the patient developed Grade 1 cytokine release syndrome (CRS) (Penn scale (16)), characterized by a peak temperature of 40.2 °C (**Figure 2**). By Day +4, the condition progressed to Grade 2 CRS, manifested by fluid-responsive hypotension, which was managed with intravenous dexamethasone (10mg q12h). Following successful defervescence, the steroid dose was tapered after three days of stability.

On Day +11, the patient experienced a secondary febrile peak accompanied by a transient decline in blood pressure, meeting the criteria for Grade 2 CRS. This episode was characterized by a profound inflammatory surge, including massive hyperferritinemia (25,500 ng/mL), hypofibrinogenemia (0.7 g/L), and markedly elevated IFN-γ (141.2 pg/mL), alongside transaminitis (ALT 122 U/L, AST 73 U/L, T-Bil 27.8 umol/L, D-Bil 14.8 umol/L).Given the high clinical suspicion for immune effector cell-associated HLH-like syndrome (IEC-HS), dexamethasone was discontinued and escalated to methylprednisolone (60 mg q12h). Concurrently, a preemptive single dose of emapalumab (1 mg/kg) was administered. This targeted intervention resulted in rapid defervescence—obviating the need for conventional antipyretics—and was followed by the steady normalization of liver function and IFN-γ levels.

**Figure 3:**
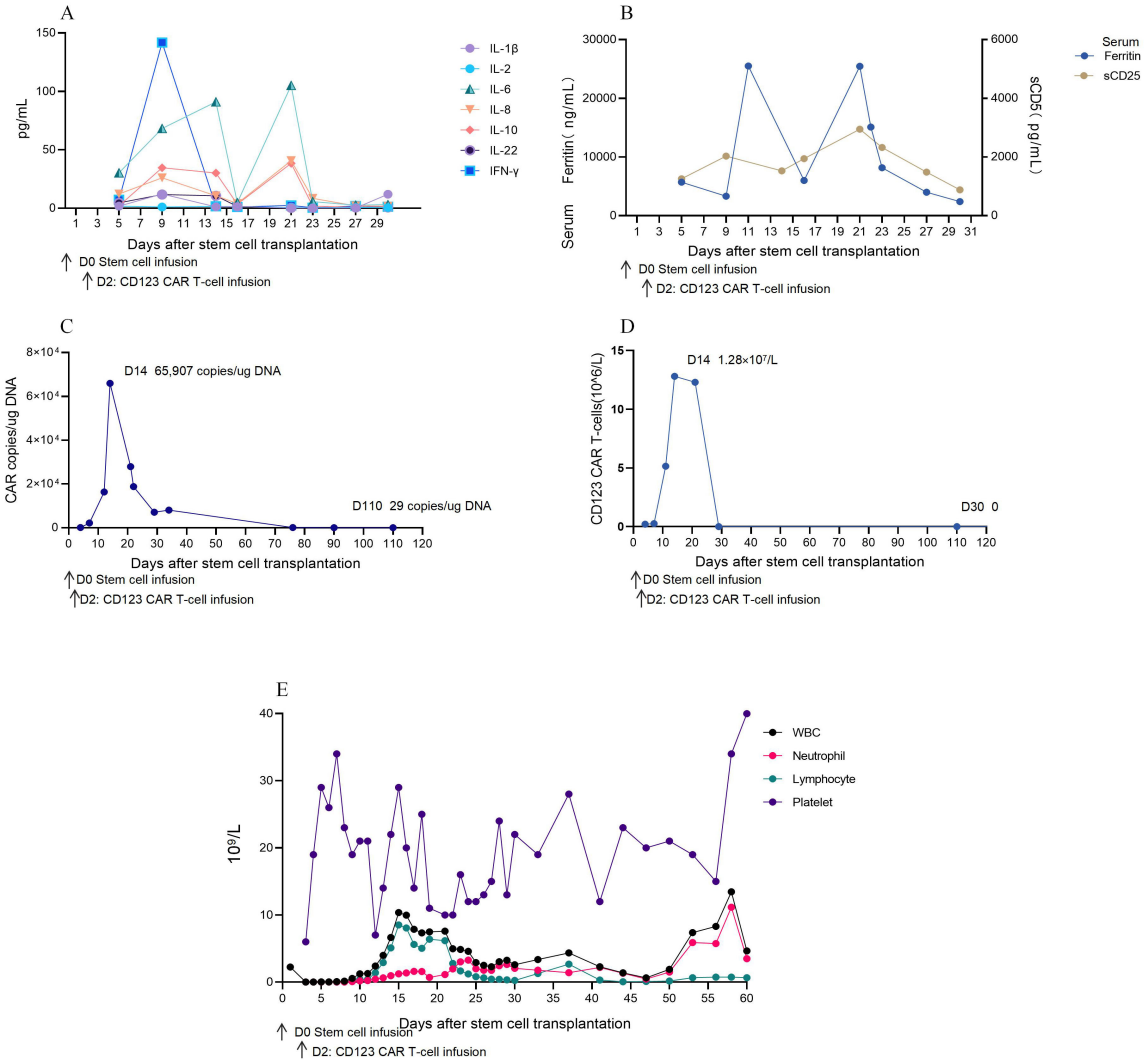
**(A)** Dynamic changes in serum cytokine profiles following CAR T-cell infusion. **(B)** Kinetics of serum ferritin and soluble CD25 (SCD25) levels post-therapy. **(C)** Kinetics of CD123 CAR T-cell expansion in peripheral blood assessed by qPCR. **(D)** Expansion of CD123 CAR T-cells in peripheral blood monitored by flow cytometry. **(E)** Kinetics of peripheral blood cell counts post-transplant.

The patient experienced a clinical rebound of CRS on Day +20, characterized by recurrent high fever and hypotension. Despite aggressive fluid resuscitation, the hypotension remained refractory, necessitating norepinephrine for hemodynamic stabilization, consistent with Grade 3 CRS. Notably, the absolute neutrophil count (ANC) concomitantly decreased to 0.7 ×10^9^/L. Laboratory evaluation revealed the following cytokine and inflammatory profile: IL-6 105.13 pg/mL, IL-8 40.64 pg/mL, IL-10 38.39 pg/mL, ferritin 25,451 ng/mL, soluble CD25 (sCD25) 2,949 pg/mL, and fibrinogen 1.3 g/L. Given the high suspicion for recurrent IEC-HS, a second dose of emapalumab (1 mg/kg) was administered. The patient responded promptly with stabilization of temperature and blood pressure. Norepinephrine was successfully discontinued on Day +25, followed by a gradual steroid taper. The methylprednisolone dose was reduced to 20 mg q12h on Day +27 and completely discontinued by Day +60. Detailed laboratory parameters fluctuations are illustrated in **Figures 3A, B**; **Supplementary Table 2**.

### 2.5 CAR-T kinetics and hematopoietic recovery

CD123 CAR T-cell expansion in the peripheral blood peaked at Day +14, reaching 65,907 copies/ug DNA via qPCR and 1.28×10^7^/L by flow cytometry. While CAR T-cell levels declined significantly after Day +30, they remained detectable until Day +110 post-infusion by qPCR (**Figures 3C, D**).

Neutrophil engraftment occurred on day +13; however, prolonged thrombocytopenia persisted, likely due to CAR-T-mediated myelosuppression. On day +37, the patient received lymphodepletion (cyclophosphamide 300mg/m^2^/d, days -4 to -2, fludarabine 30 mg/m²/d, days -4 to -3, and etoposide 50mg/d, days -4 to -2) to eliminate residual CAR-T cells, followed by a second “rescue” infusion of stem cells (CD34+cell 1.0×10^6^/kg). Platelet engraftment was successfully achieved by day +58 (**Figure 3E**).

Standard antimicrobial prophylaxis was maintained throughout, with no documented opportunistic infections. During the period of absolute neutropenia (ANC < 0.5 ×10^9^/L), the patient received prophylactic antibacterial, antifungal, and antiviral therapies. Prophylactic antibiotics were discontinued upon successful neutrophil engraftment. In contrast, due to the prolonged use of high-dose corticosteroids, prophylactic antifungal therapy was strictly maintained throughout the steroid course and was discontinued two weeks after the complete cessation of corticosteroids, as the patient remained afebrile with no clinical signs of infection. Antiviral prophylaxis was maintained until the CD4+ T-cell count recovered to > 200/uL. Additionally, sulfamethoxazole/trimethoprim was administered for one year post-transplant for Pneumocystis jirovecii pneumonia (PJP) prophylaxis.

### 2.6 Maintenance and follow-up

**Figure 4:**
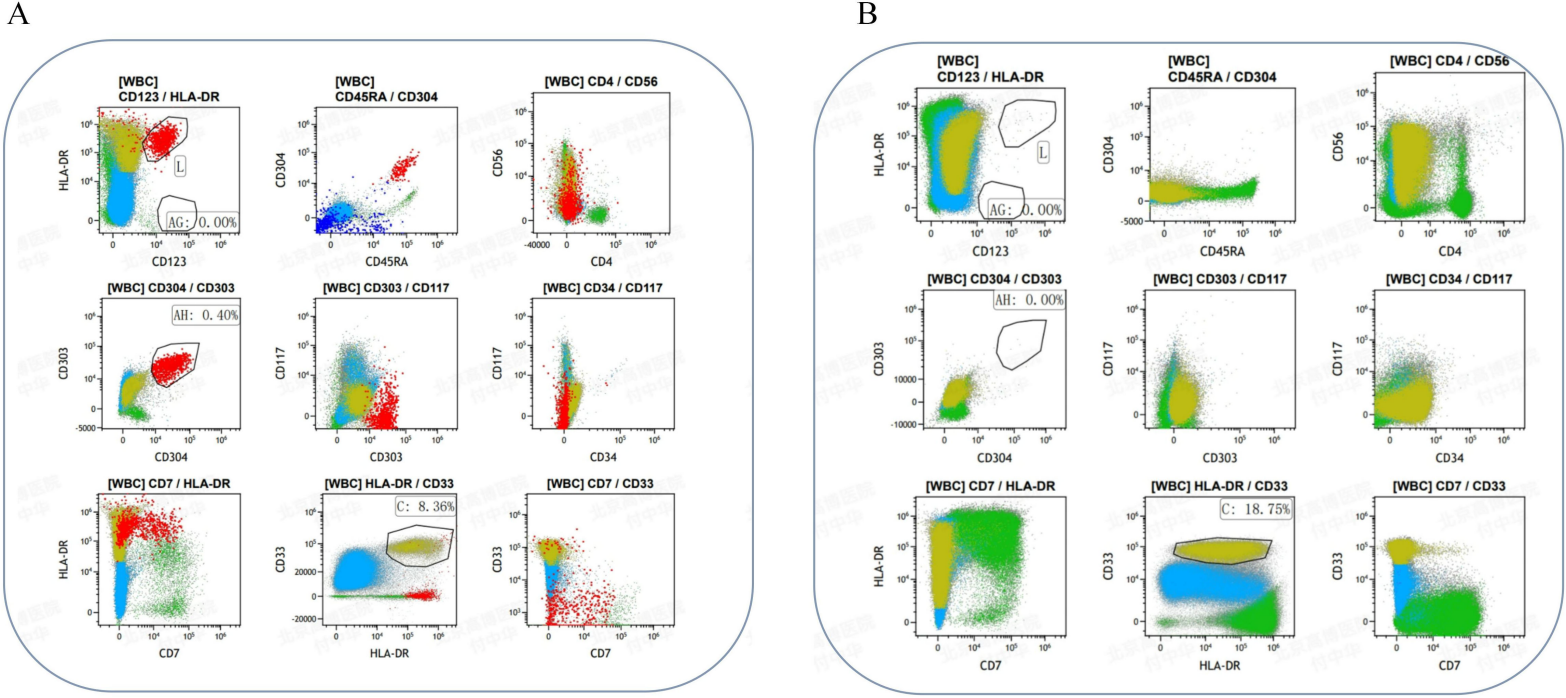
**(A)** Flow cytometry analysis before ASCT combined with CD123 CAR-T therapy. Abnormal phenotypic pDC cells account for 0.40% of total cells, expressing:CD123dim, HLADR, CD304, CD303, CD45RA, CD4dim, CD56part, CD7part, CD33part; CD117(-), CD34(-). **(B)** Flow cytometry analysis at day +25 post ASCT combined with CD123 CAR-T therapy.No abnormal phenotypic pDC cells were detected.

The patient achieved early molecular clearance, with BM flow cytometry showing no evidence of MRD on Days +18 and +25 (**Figure 4**). Full hematological recovery and persistent MRD negativity in both bone marrow and cerebrospinal fluid (CSF) were documented at Day +90.Maintenance therapy with Venetoclax (50 mg/day) was initiated at month 3 post-transplant. At the 13-month follow-up, the patient remains in sustained CR, demonstrating persistent MRD-negative status in both BM and CSF, as well as a complete response on PET-CT.

## 3 Discussion

The management of relapsed/refractory (R/R) BPDCN, particularly in patients lacking suitable allogeneic donors, remains a critical unmet medical need. Our patient presented with characteristic “systemic plus skin” involvement, featuring the diagnostic triad of CD123, CD4, and CD56 expression (18–25).Despite achieving a complete metabolic response (CMR) and CNS clearance after nine cycles of Hyper-CVAD, the persistence of bone marrow (BM) minimal residual disease (MRD) remained a major concern. Based on prognostic models factoring in age (≥50 years) and overt BM involvement, this patient fell into the intermediate-to-adverse risk category, with a dismal estimated 2-year overall survival (OS) of 5%–39% (18, 26, 27).While frontline intensive chemotherapy followed by allogeneic hematopoietic stem cell transplantation (allo-HSCT) can lead to durable remissions (4, 5), this consolidation strategy was unfeasible for our patient due to the absence of a human leukocyte antigen (HLA)-matched or haploidentical donor. Furthermore, although novel targeted therapies like the CD123-directed cytotoxin tagraxofusp and the antibody-drug conjugate pivekimab sunirine (PVEK) have demonstrated promising efficacy in both treatment-naïve and R/R settings (6, 28), these agents are currently inaccessible in China. Given the high likelihood of imminent relapse due to persistent MRD and the lack of standard salvage options, an alternative therapeutic approach was urgently required.

Emerging data from early-phase clinical trials have demonstrated the potent anti-leukemic activity of anti-CD123 CAR T-cells in BPDCN; however, several formidable hurdles persist. A primary limitation is the restricted long-term persistence and inconsistent *in vivo* expansion, which often leads to early relapse in the absence of further consolidation (29–31). Furthermore, since CD123 is also expressed on normal hematopoietic stem and progenitor cells (HSPCs), “off-tumor” myelosuppression and prolonged, life-threatening cytopenias remain critical safety barriers (1, 32). To address these challenges, integrating autologous stem cell transplantation (ASCT) with CAR-T therapy offers a dual-purpose solution: the high-dose conditioning regimen facilitates a more favorable niche for CAR-T expansion through homeostatic cytokine induction, while the subsequent infusion of autologous stem cells provides a vital “hematopoietic rescue” to mitigate the expected myeloid toxicity.

### 3.1 Synergistic rationale for ASCT combined with CD123 CAR-T

While allogeneic HSCT is the preferred consolidation for BPDCN, autologous HSCT (ASCT) has shown promising results in CR1 patients, with 4-year OS rates reaching 82% in certain cohorts (33). However, the risk of relapse due to graft contamination or residual chemo-resistant clones remains a significant limitation (7).

Integrating CD123-targeted CAR-T therapy into the ASCT framework addresses these challenges through several mechanisms. First, ASCT-mediated lymphodepletion and cytoreduction reshape the immunosuppressive tumor microenvironment (TME), potentially enhancing CD123 CAR-T expansion and persistence (34–36). Second, CAR-T cells act as an *in vivo* “purging” agent to eradicate residual MRD and prevent graft-related recurrence (37). Given the initial CNS involvement in our patient, the established efficacy of sequential ASCT and CAR-T therapy in CNS lymphoma suggests that this integrated approach likely facilitated the eradication of residual CNS disease and provided sustained neuro-immunosurveillance (38).

A major hurdle for CD123 CAR-T is “on-target/off-tumor” myelosuppression, as CD123 is expressed on normal hematopoietic stem cells (HSCs) (39). In this case, ASCT functioned uniquely as a “hematopoietic rescue.” Although CAR-T expansion led to delayed platelet recovery—likely due to prolonged immune-mediated suppression—the subsequent infusion of backup autologous stem cells facilitated successful engraftment by day +58. This suggests that ASCT sequential CAR-T is not only efficacious but offers a critical safety net against prolonged cytopenias.

### 3.2 Management of immune-related toxicities

The clinical course was complicated by suspected immune effector cell-associated HLH-like syndrome (IEC-HS), characterized by extreme hyperferritinemia (>10,000 ng/mL), hypofibrinogenemia, and elevated IFN-γ (40). Given the high mortality associated with refractory IEC-HS, we preemptively administered emapalumab (an anti-IFN-γmonoclonal antibody), which led to rapid stabilization (41). Although there are currently no established guidelines for the prophylactic or preemptive use of emapalumab in IEC-HS, we administered a single dose (1 mg/kg) during each episode based on the patient’s clinical status, which resulted in remarkable therapeutic efficacy. This highlights the necessity of vigilant monitoring for cytokine-mediated toxicities beyond standard CRS and the potential role of targeted neutralizers in managing severe CAR-T-related complications.

### 3.3 Maintenance therapy and long-term outcomes

Given the high relapse risk in R/R BPDCN, we initiated maintenance with venetoclax, a BCL-2 inhibitor. BPDCN exhibits an exquisite dependency on BCL-2, and venetoclax has demonstrated potent preclinical and clinical activity in this setting (42–44). The convenience of oral administration and the patient’s sustained 13-month disease-free survival (DFS) suggest that venetoclax may play a decisive role in preventing “late-escape” of residual clones post-transplant.

## 4 Conclusion

For R/R BPDCN patients lacking an allogeneic donor, the sequential application of ASCT and anti-CD123 CAR-T cells, followed by venetoclax maintenance, suggests a potentially feasible management approach. This “triad” strategy—combining intensive consolidation, targeted immunotherapy, and molecular maintenance—holds promise for addressing the therapeutic challenges in this high-risk population. However, given the inherent limitations of a single case evaluation, further robust clinical studies are strictly required to establish its broader applicability and safety.

## Data Availability

The original contributions presented in the study are included in the article/**Supplementary Material**. Further inquiries can be directed to the corresponding author.
