# Supplementary material for: Anti-CD123 CAR-T therapy combined with autologous SCT and venetoclax maintenance in refractory BPDCN ineligible for allogeneic transplantation: a case report and review of the literature
Source: Front Immunol. 2026 Jul 31;17:1922400. doi: 10.3389/fimmu.2026.1922400 (PMC13473410; doi:10.3389/fimmu.2026.1922400)
Supplement: Supplementary file 1 [file Table1.docx]

Suppl.table 1. Clinical and Laboratory Assessments at Baseline and Prior to ASCT/CAR T-Cell Therapy

|  |  | **Before initial treatment** | **Before ASCT combined with CAR-T therapy** |
| --- | --- | --- | --- |
| Blood cell count | | WBC 2.6 ×10^9/L  Neut 1.58×10^9/L  Lym 1.1×10^9/L  HGB 96g/L  PLT 141 ×10^9/L | WBC 2.83 ×10^9/L  Neut 1.07×10^9/L  Lym 1.43×10^9/L  HGB 124g/L  PLT 106×10^9/L |
| Biochemical and Coagulation Parameters | | Normal | Normal |
| BM | Morphological analysis | Bone marrow hyperplasia grade II. Dendritic cells account for 26%, showing:  - Variable cell size, round to slightly indented nuclei, faintly visible nucleoli, moderate gray-blue cytoplasm, and partial vacuolar degeneration.  - Leukemia cytochemical staining:  - MPO: 89% positive, 11% weakly positive  - NSE: 100% positive  - NF: 100% positive  - PAS: 100% positive | Markedly hypercellular marrow. Myeloid series proportion is approximately normal (40.5%), with myelocytes and earlier stages present and no significant morphologic abnormalities |
|  | Flow cytometry analysis | Abnormal phenotypic pDC cells account for 2.66% of nucleated cells. Phenotype:CD123+，HLA-DR+，BDCA-2-，BDCA-4+，CD56+，CD4dim+，CD14-，CD33- | Abnormal phenotypic pDC cells account for 0.40% of total cells, expressing:CD123dim，HLADR，CD304，CD303，CD45RA，CD4dim，CD56part，CD7part，CD33part; CD117(-)，CD34(-) |
|  | G-banding chromosome karyotyping analysis | 46.XY[20] | Not tested |
|  | Next-generation sequencing [NGS] | Not tested | Not tested |
| CSF | Flow cytometry analysis | Abnormal phenotypic pDC cells account for 10.54% of total cells, expressing:CD123+，HLA-DRdim+、BDCA-4+、BDCA-2+、CD56+、CD4dim+、CD14- | (-) |
| PET/CT | | Multiple systemic FDG-avid lesions involving upper/lower lymph nodes, liver, spleen, bone marrow, and skin, consistent with blastic plasmacytoid dendritic cell neoplasm (BPDCN) | Complete metabolic response  (CMR) |
| Enhanced MRI of the head | | Normal | Normal |
| Electrocardiogram (ECG/EKG) | | Normal | Normal |
| Echocardiography | | Normal ejection fraction (EF) | Normal ejection fraction (EF) |
| ECOG PS | | 1 | 1 |
