# Supplementary material for: Anti-CD123 CAR-T therapy combined with autologous SCT and venetoclax maintenance in refractory BPDCN ineligible for allogeneic transplantation: a case report and review of the literature
Source: Front Immunol. 2026 Jul 31;17:1922400. doi: 10.3389/fimmu.2026.1922400 (PMC13473410; doi:10.3389/fimmu.2026.1922400)
Supplement: Supplementary file 2 [file Table2.docx]

Suppl. table 2. Kinetics of Serum Biochemistry and Coagulation Profiles Within 30 Days Post-Transplant

| **Item** | **0** | **+2** | **+3** | **+4** | **+5** | **+7** | **+9** | **+10** | **+11** | **+13** |  | **+14** | **+15** | **+16** | **+18** | **+21** | **+22** | **+23** | **+25** | **+27** | **+30** |
| --- | --- | --- | --- | --- | --- | --- | --- | --- | --- | --- | --- | --- | --- | --- | --- | --- | --- | --- | --- | --- | --- |
|  | Stem  cell  infusion | CAR-T  Cell   infusion |  |  |  |  |  |  |  |  |  |  |  |  |  |  |  |  |  |  |  |
| ALT  (U/L) | 44 | 40 | 37 | 41 | 78 | 56 | 44 | 46 | 122 | 122 |  | 86 | 85 | 63 | 93 | 37 | 31 | 51 | 115 | 110 | 32 |
| AST  (U/L) | 28 | 24 | 20 | 30 | 42 | 14 | 14 | 18 | 73 | 41 |  | 31 | 25 | 16 | 26 | 37 | 20 | 28 | 34 | 12 | 24 |
| TBIL  (μmol/L) | 13.2 | 14.7 |  | 12.4 | 13.3 | 17 | 29.9 | 30 | 27.8 | 24.8 |  | 21.6 | 19.9 | 18.2 | 16.6 | 12.2 | 11.1 | 12.7 | 14.6 | 14.7 | 18 |
| DBIL  (μmol/L) | 3.7 | 4.1 |  | 4.4 | 4.4 | 5.8 | 10.5 | 12.5 | 14.8 | 11.8 |  | 10.6 | 8.9 | 8.6 | 7.6 | 5.5 | 4.4 | 5.4 | 5.2 | 5.1 | 7.4 |
| SCr  (μmol/L) | 64 | 67.8 | 55.9 | 63.7 | 49.5 | 55.7 | 49.9 |  | 43.7 | 48.7 |  | 48.7 | 41.5 | 38 | 40.5 | 39.4 | 36.6 | 37.5 | 26.9 | 28.1 | 32 |
| PT  (S) |  | 11.4 |  | 12.9 | 12 | 13 | 12.2 |  | 16 |  |  |  |  | 13.5 |  | 16.7 |  | 13.1 |  | 12.7 | 12.2 |
| APTT  (S) |  | 27.9 |  | 30.6 | 31.1 | 29.8 | 29.4 |  | 49.1 |  |  |  |  | 41.1 |  | 49 |  | 35.8 |  | 27.4 | 25 |
| PTA% |  | 95 |  | 70.70 | 83.70 | 83.70 | 80.40 |  | 48.20 |  |  |  |  | 64.90 |  | 45.10 |  | 68.70 |  | 72.80 | 84.2 |

Abbreviation：

ALT: Alanine Aminotransferase; AST: Aspartate Aminotransferase; TBIL: Total Bilirubin; DBIL: Direct Bilirubin; SCr: Serum creatinine; PT: Prothrombin Time; APTT: Activated Partial Thromboplastin Time; PTA: Prothrombin Activity;
